# Les dysthyroïdies chez l’hémodialysé chronique

**Published:** 2010-10-07

**Authors:** Zbiti Najoua, Hakima RhouZbiti, Fatima Ezaitouni, Naima Ouzeddoune, Rabia Bayahia, Loubna Benamar

**Affiliations:** 1 Service de Néphrologie-Dialyse-Transplantation rénale, CHU Ibn Sina, Rabat, Maroc

**Keywords:** Hémodialyse chronique, hypothyroïdie, facteurs prédictifs, Maroc

## Abstract

**Introduction:**

Les dysthyroïdies chez l’hémodialysé chronique sont représentées par une hypothyroïdie: dite le syndrome de basse T3, actuellement nommée “le syndrome de la maladie euthyroïdienne”. L’objectif de notre travail est de déterminer le profil thyroïdien de nos HDC afin de préciser la prévalence des différents troubles thyroïdiens dans notre population et de dégager les facteurs prédictifs, aussi on a précisé l’évolution sur une période de 3 ans.

**Méthodes:**

Il s’agit d’une étude rétrospective réalisée en Mars 2007 concernant 68 patients adultes en hémodialyse périodique. Nous avons analysé les paramètres anthropométriques, cliniques et biologiques. Le dosage des hormones thyroïdiennes ((triiodothyronine libre) FT3, (thyroxine libre) FT4 et (thyréostimuline ultra-sensible) TSHus) s’est effectué selon la méthode radio-immunologique. Nous avons comparé 2 groupes de patients avec et sans dysthyroïdie (définie par un taux anormal de FT3 et/ou de FT4 et/ou de TSH) afin de dégager les facteurs de risque. On a réalisé une surveillance biologique des hormones thyroïdiennes pendant 3 ans afin de préciser l’incidence des différents troubles thyroïdiens dans la même population.

**Résultats:**

Tous les patients sont en euthyroïdie clinique. On n’a noté aucun cas de goitre. Le profil thyroïdien est caractérisé par une hypothyroïdie biologique, la prévalence était de 28%. Nous n'avons pas trouvé des cas d'hyperthyroïdie. L'étude comparative des deux groupes de malades biologiquement euthyroïdiens et hypothyroïdiens a révèle le syndrome inflammatoire (p=0.001), l’âge avancé (p=0.003), et la durée prolongée en hémodialyse (p=0.04) comme des facteurs de risque liés à l’hypothyroïdie biologique. Deux HDC ont nécessité un traitement hormonal substitutif vu le contexte du diabète dans un cas et de la grossesse dans l’autre cas. Le suivi biologique pendant trois ans n’a révélé aucun cas incident et aucune aggravation.

**Conclusion:**

L’hypothyroïdie biologique est un trouble endocrinien fréquent en hémodialyse chronique. L’âge avancé et le syndrome inflammatoire semblent être des facteurs de risque.

## Introduction

Les anomalies thyroïdiennes chez l’hémodialysé chronique (HDC) sont secondaires à des troubles hormonaux, ils sont représentés par une hypothyroïdie biologique, exceptionnellement par une hyperthyroïdie [1]. L’hypothyroïdie dans ce cas est primaire, elle est définie par une euthyroïdie clinique associée à une hypothyroïdie biologique: 1) la Triiodothyronine libre (FT3) est souvent basse, 2) la Thyroxine libre (FT4) est souvent normale, 3) la thyréostimuline ultra-sensible (TSHus) peut être élevée [2].

L’hormone FT3 est l’hormone biologiquement active, elle est souvent basse chez l’HDC, ceci traduit « Le syndrome de basse T3 [3]. Ce dysfonctionnement endocrinien est grave, puisqu’il représente un puissant facteur de risque de morbi-mortalité cardio-vasculaire [4].

La physiopathologie reste insuffisamment élucidée; une évidence récente met en évidence le rôle majeur du syndrome inflammatoire [5]. Les données de la littérature sont pauvres. Il n’existe ni consensus ni recommandations pouvant éclaircir et faciliter la prise en charge thérapeutique des troubles thyroïdiens chez le dialysé. Le but de notre travail est de déterminer le profil thyroïdien de nos HDC afin de préciser la prévalence et l’incidence, de dégager les facteurs prédictifs liés à chaque trouble thyroïdien et de préciser l’évolution sur une période de 3 ans.

## Méthodes

Il s’agit d’une étude rétrospective réalisée en Mars 2007 incluant 68 patients adultes en HDC ayant un âge supérieur à 18 ans. On a exclu tout patient recevant un traitement pouvant interférer avec la fonction thyroïdienne (les anti-thyroïdiens, le lithium, l’amiodarone, la rifampicine).

**L’évaluation de la dialyse**

Nous nous sommes intéressés aux paramètres anthropométriques (l’âge, le sexe), aux facteurs de risque cardiovasculaire (l’âge > 50 ans, le diabète, l’hypertension artérielle (HTA), l’excès pondéral et le tabac), au déroulement des séances de dialyse et aux critères de dialyse adéquate: le Kt/V, le pourcentage de réduction de l’urée (PRU), l’état nutritionnel et inflammatoire (le poids sec, l’indice de masse corporelle (IMC) et l’albuminémie en pré-dialyse, la protéine C réactive (CRP); l’équilibre phosphocalcique (la calcémie, la phosphorémie, les phosphatases alcalines osseuses, la parathormone intacte (PTH1-84), la réserve alcaline (RA); l’anémie (l’hémoglobinémie, la férritinémie).

**L’évaluation endocrinienne**

Nous avons effectué un examen clinique systématique à la recherche de signes dysthyroïdiens: d’hyperthyroïdie ou d’hypothyroïdie (prise de poids, asthénie, frilosité , bradycardie, trouble de transit digestif (diarrhée, constipation), apathie, somnolence et des signes de thyrotoxicose). Nous avons réalisé un examen clinique de la loge thyroïdienne complété par une échographie cervicale à la recherche de goitre. Les prélèvements sanguins ont été faits avant le branchement des malades en dialyse. Nous avons dosé le taux sanguin des hormones thyroïdiennes: La FT3, la FT4 par la méthode radio-immunologique selon le principe de compétition en utilisant un anticorps marqué; et la TSHus selon la méthode immunoradiométrique de type sandwich. Les valeurs de référence considérées pour les hormones thyroïdiennes sont: FT3 = 1,6 - 3,8 pg/ml; FT4 = 9 - 18 pg/ml; TSHus = 0,2 - 4 Zg/ml.

Nous avons comparé deux groupes de patients avec et sans dysthyroïdie afin de dégager les facteurs de risque. Nous avons mené une confrontation néphrologique et endocrinologique des dossiers des HDC dans le but de discuter et de décider de façon sage et réfléchie la démarche thérapeutique.

**L’analyse statistique**

Elle s’est fondée sur le logiciel statistique SPSS 11.0. Nous avons utilisé pour l’analyse des variables quantitatives: la moyenne ± écart-type; et pour les variables qualitatives: le pourcentage. Nous avons utilisé le test t de Student pour la comparaison des moyennes et le chi-2 pour les pourcentages. La différence a été considérée comme significative quand le p était inférieur à 0,05. On a complété l’analyse univariée par l’analyse multivariée afin de dégager les facteurs de risques liés à ces anomalies thyroïdiennes.

## Résultats

**Figure 1:**
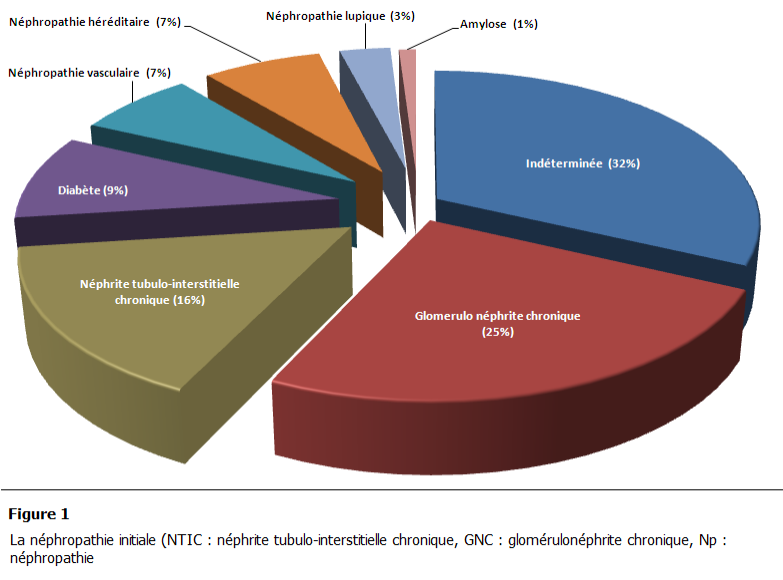
La néphropathie initiale (NTIC: néphrite tubulo-interstitielle chronique, GNC: glomérulonéphrite chronique, Np: néphropathie.

Notre série comprend 68 patients HDC adultes ayant un âge supérieur à 18 ans. L’âge moyen était de 43,4 ± 13,5 ans (les extrêmes étaient de 18ans et 72ans). On a noté une nette prédominance féminine (42 femmes et 26 hommes). La durée hebdomadaire de dialyse était de 12 heures avec une durée totale moyenne en hémodialyse de 113 ± 58 mois. La néphropathie initiale était dominée par une origine glomérulaire (notée dans 25 % des cas), le diabète représentait 9% des cas, l’origine tubulo-interstitielle a été notée chez 16 % des patients (Figure 1).

L’analyse des paramètres biologiques a retrouvé une hémoglobine moyenne de 9,1 ± 2,3 g/dl, avec une férritinémie moyenne de 432 ± 414 Zg/ml. Rares sont les malades qui recevaient un traitement de l’anémie à base d’érythropoïétine recombinante (22% des cas). La plupart ont été mis sous fer injectable et/ou oral. Les troubles phospho-calciques ont été dominés par une hyperparathyroïdie avec une PTH1-84 moyenne de 643,4 ± 593 pg/ml, une calcémie moyenne de 89 ± 9 mg/l, une phosphorémie moyenne de 52 ± 12 mg/l, une acidose avec une RA moyenne de 19 ± 2 mEq/l, un taux élevé de PAL et un produit phospho-calcique très élevé. On a noté une qualité de dialyse caractérisée par un Kt/V moyen de 1,58 ± 0,3 et un PRU à 77,7 ± 6,5 %. L’état inflammatoire et nutritionnel ont été caractérisés par une CRP moyenne de 9,2 ± 5,5 mg/l et une albuminémie pré-dialytique moyenne de 37,8 ± 4 g/l (Tableau 1).

**Le profil thyroïdien**

Sur le plan clinique, tous les patients HDC étaient en euthyroïdie clinique. L’examen échographique était normal sans image de goitre ni de nodule.

**Figure 2:**
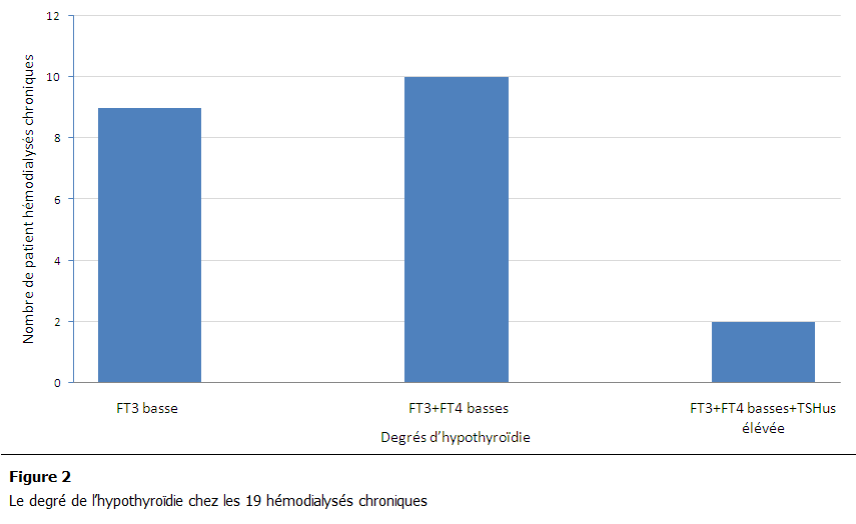
le degré de l’hypothyroïdie chez les 19 hémodialysés chroniques

Sur le plan biologique, le dosage hormonal a révélé une hypothyroïdie chez 19 patients soit une prévalence estimée à 28%. FT3 était basse chez 19 patients avec une moyenne de 1 ± 0,26 pg/ml, 10 patients avaient à la fois une baisse de FT3 et de FT4, avec une FT4 moyenne de 7,5 ± 1 pg/ml, 2 parmi les HDC avec FT3 et FT4 basses avaient une TSHus très élevée dépassant les 50 Zg/ml (Figure 2).

Comparaison des deux groupes avec et sans hypothyroïdie

L’analyse uni-variée a démontré que l’âge avancé (> 50 ans), le syndrome inflammatoire et la durée prolongée en hémodialyse étaient des facteurs de risque liés à l’hypothyroïdie biologique chez l’HDC (Tableau 2). En analyse multi-variée, seuls l’âge avancé (> 50 ans) et le syndrome inflammatoire représentaient des facteurs de risque indépendants de l’hypothyroïdie biologique chez l’HDC.

**La prise en charge thérapeutique**

L’hypothyroïdie biologique a été associée à un diabète dans un premier cas et à une grossesse à 28 semaines d’aménorrhée chez une seconde malade. Une confrontation néphrologique et endocrinologique a été menée afin de discuter la démarche thérapeutique: seules les deux patientes ayant un retentissement sur l’axe hypothalamo-hypophysaire avec une TSHus élevée ont bénéficié d’un traitement substitutif hormonal à base de la L-thyroxine (LEVOTHYROX®), à doses progressives. La dose journalière initiale était de 50 µg. Pour le reste des patients en hypothyroïdie biologique (au nombre de 17), on a opté pour une abstention thérapeutique associée à une stricte surveillance clinique et biologique.

**Le caractère évolutif**

Chez la patiente enceinte: l’accouchement s’est déroulé par césarienne, donnant naissance à un nouveau-né de sexe féminin sans souffrance néonatale, le poids à la naissance était de 3100 g, il n’y avait pas de signes d’hypothyroïdie néonatale. Une réduction progressive de L-thyroxine s’est imposée chez la maman devant une baisse du taux de TSHus sans pouvoir l’arrêter vue une réascension du taux de TSHus. Quant à la seconde patiente diabétique: la L-thyroxine a été maintenue avec une stricte surveillance clinique et biologique. Nous n’avons noté aucun problème cardio-vasculaire compliquant la substitution hormonale.

Depuis la réalisation de l’étude, un bilan thyroïdien annuel est systématiquement réalisé chez tous les HDC du centre. Nous n’avons noté aucun cas incident de dysthyroïdie durant les trois années de suivi, et aucune aggravation biologique n’a été remarquée: En 2008: deux patients (en euthyroïdie clinique et biologique) sont décédés suite à une pneumopathie virale grave compliquée d’un choc septique chez le premier et à un infarctus du myocarde dans le deuxième cas; en 2009: deux patients (en euthyroïdie clinique et biologique) sont décédés suite à un sepsis à staphylocoque à point de départ la voie d’abord chez le premier et à un infarctus du myocarde dans le second cas; en 2010: un HDC âgé de 80 ans (ayant plusieurs facteurs de risque cardio-vasculaires associés à une baisse isolée de FT3: syndrome de basse T3) est décédé suite à une dénutrition sévère (Tableau 3).

## Discussion

Au stade embryonnaire, il existe une relation étroite entre la glande thyroïde et les reins. Les hormones thyroïdiennes jouent un rôle important dans le développement et la physiologie du rein; ce dernier est, à son tour, nécessaire pour la sécrétion, le métabolisme et l’élimination des hormones thyroïdiennes [6-8].

**Figure 3:**
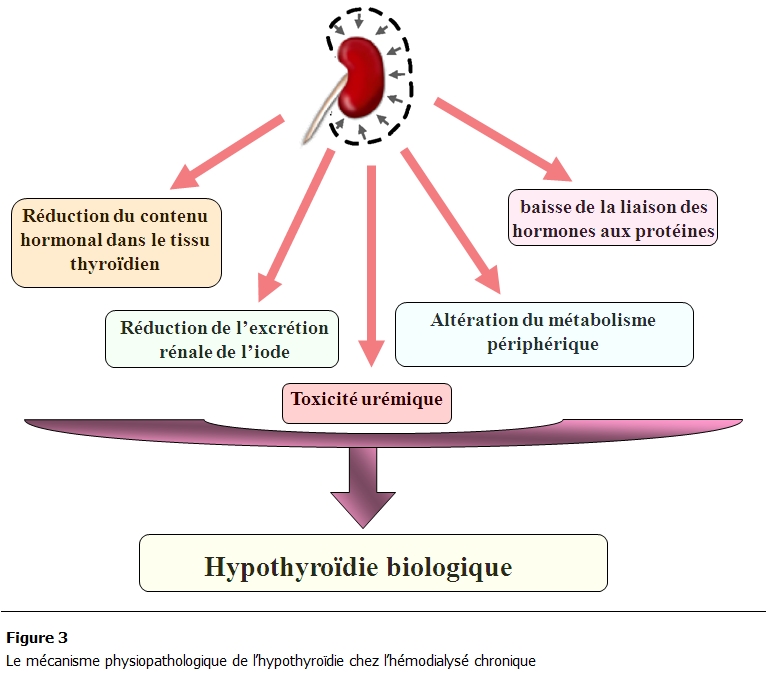
le degré de l’hypothyroïdie chez les 19 hémodialysés chroniques

Tout dysfonctionnement thyroïdien risque d’engendrer des perturbations hémodynamiques et vasculaires sur le glomérule, la fonction tubulaire et la balance hydro-sodée ainsi que des troubles électrolytiques notamment des dysnatrémies [6,9]. Toute altération de la fonction rénale retentit sur l’axe hypothalamo-hypophysaire et le métabolisme périphérique des hormones thyroïdiennes [3,6,10] (Figure 3).

Le volume de la glande thyroïdienne augmente chez les patients insuffisants rénaux chroniques. Le goitre est fréquemment retrouvé dans cette population avec une prédominance féminine [1,5,9,11-13]. Dans notre série, l’examen clinico-échographique n’a objectivé aucun cas de goitre.

La physiopathologie des troubles thyroïdiens associés à une insuffisance rénale chronique n’est pas bien élucidée [14]. Des études réalisées entre les années 1980 et 1990 ont montré qu’ils seraient secondaires à une perturbation du métabolisme périphérique des hormones par la réduction de la conversion périphérique de FT4 en FT3 du fait de la diminution de l’activité de 5’-déiodinase de type I, à la réduction de la liaison des hormones thyroïdiennes à la thyroglobuline et à l’accumulation d’un iode inorganique secondaire à la diminution de l’excrétion rénale de l’iode [4,9], l’acidose serait aussi, incriminée dans cette diminution de la synthèse périphérique des hormones thyroïdiennes [15].

L’anticoagulation per-dialytique à base de l’héparine empêche la liaison de FT4 aux protéines avec un taux faussement augmenté. Nous avons réalisé tous les prélèvements avant le branchement des malades pour la dialyse.

Récemment, il a été démontré que le taux des hormones thyroïdiennes diminue avec la diminution du débit de filtration glomérulaire (DFG); la prévalence de l’hypothyroïdie passe de 7 % si le DFG b 90 ml/min à 17,9 % si le DFG < 60ml/min [14]. Elle est plus fréquente chez les femmes et souvent associée à un taux élevé des anticorps anti thyroïde [3].

En effet, le trouble thyroïdien le plus fréquent chez l’HDC est l’hypothyroïdie primaire [6,16]. La réduction de FT3 (le syndrome de basse T3) est fréquemment observée chez les dialysés avec une euthyroïdie clinique [1,3,9,16]. La prévalence de l’hypothyroïdie primaire varie de 9% à 25% des cas. Rares sont les équipes qui se sont intéressées à l’ origine auto-immune [14,16,17]. La prévalence de l’hypothyroïdie biologique dans notre série est de 28% (la baisse isolée de FT3 a été retrouvée dans 9 cas soit 13 % des cas). Un dosage des anticorps anti thyroglobuline et anti peroxydase serait d’intérêt étiologique important.

L’hyperthyroïdie est exceptionnellement retrouvée chez l’HDC. La prévalence est similaire à celle retrouvée dans la population générale (environ 1% de la population), elle est considérée comme une cause de résistance de l’anémie à l’érythropoïétine recombinante. Dans notre série, nous n’avons noté aucun cas d’hyperthyroïdie.

Le complexe malnutrition-inflammation est un problème clinique majeur chez les dialysés [18]. En effet, les cytokines réduisent la circulation des hormones thyroïdiennes. Une réduction plasmatique de FT3 (l’hormone thyroïdienne biologiquement active) est le dysfonctionnement thyroïdien le plus précoce chez l’HDC, qui sera suivi d’une baisse de FT4 [5,18-20]. Il est intéressant de noter la relation entre l’état inflammatoire chez l’insuffisante rénale chronique et le degré d’une dysfonction endothéliale [3,5]. La baisse de FT3 est souvent associée à un état inflammatoire chronique, elle est indépendamment associée à une forte mortalité cardio-vasculaire [4,5,13,21-24], c’est un facteur prédicteur de mortalité en hémodialyse [25,26]. La relation entre l’inflammation et le dysfonctionnement thyroïdien reste complexe et peut être bidirectionnelle. En effet, la lipoprotéine a, une protéine pro-athéromateuse qui est associée fortement à l’élévation de la CRP chez l’HDC, est significativement réduite sous la L-thyroxine [4,27,28].

En dialyse péritonéale (DP), l’hypothyroïdie primitive est la plus fréquente suivie du syndrome de basse T3 avec une prévalence, respectivement, avoisinant 27,5 % et 16 % [29,30]. La perte continue de protéines dans la cavité péritonéale serait responsable d’une augmentation de l’incidence de dysthyroïdie. Ce trouble peut être impliqué dans l’atteinte cardiaque en DP car la majorité des malades ont une faible fraction d’éjection du ventricule gauche (d’où l’indication de la DP) en comparaison avec ceux ayant une TSHus normale [29]. Une fois, l’hypothyroïdie installée, la fonction ventriculaire gauche risque d’être compromise.

La baisse de FT3, est souvent associée à une durée longue en dialyse, à une acidose, à des marqueurs d’inflammation et à des marqueurs d’altération endothéliale [31]. Les résultats de notre travail avoisinent nettement ceux de la littérature, en particulier des études récentes notamment l’étude de Zoccali et ses collaborateurs (Tableau 4); avec comme facteurs de risque communs l’âge avancé et le syndrome inflammatoire.

Quand le diagnostic biologique d’une hypothyroïdie chez un HDC est établi, l’arrêt d’une cause réversible (médicament et produit de contraste iodé), doit être discuté [4,5,32]. Si l’hypothyroïdie est profonde et irréversible un traitement substitutif à base de L-Thyroxine devrait être administré [5]. Les doses doivent être ajustées afin d’obtenir des taux sériques normaux des hormones et de ne pas induire une hyperthyroïdie. Une fois l’euthyroïdie biologique est obtenue, une réduction progressive des doses de L-Thyroxine est nécessaire [5,15].

Devant l’absence d’un consensus thérapeutique actuel, une substitution hormonale a été indiquée chez nos deux patientes ayant un retentissement sur l’axe hypothalamo-hypophysaire avec une TSHus élevée; en plus dans la crainte d’une hypothyroïdie néonatale dans le cas associé à une grossesse. Des études ont démontré que le meilleur traitement reste la transplantation rénale [32], elle s’accompagne d’une amélioration de la fonction thyroïdienne [33]. Il existe une corrélation positive entre la créatinine sérique et le volume thyroïdien [32,34]. Un taux bas de FT3 avant la greffe serait associée à un risque de perte de greffon [34-37]. Le traitement hormonal ne semble pas améliorer la qualité du greffon ou prolonger la survie du greffon [38].

## Conclusion

Les anomalies endocriniennes chez le dialysé sont insuffisamment élucidées. Les données de la littérature restent pauvres. Les troubles thyroïdiens chez les patients en hémodialyse périodique sont, souvent, représentés par une hypothyroïdie biologique avec une euthyroïdie clinique: «le syndrome de la maladie euthyroïdienne». L’hormone FT3 (l’hormone biologiquement active) est souvent retrouvée basse. Ce dysfonctionnement endocrinien est grave, puisqu’il représente un puissant facteur de risque de morbi-mortalité cardio-vasculaire. L’âge avancé et le syndrome inflammatoire sont les principaux facteurs de risque liés à l’hypothyroïdie biologique. La substitution hormonale n’est pas toujours indiquée. Une stricte surveillance clinique et biologique s’avère nécessaire. Un dépistage systématique des toubles thyroïdiens chez l’HDC s’avère nécessaire, et un suivi strict annuel s’impose.

## Conflits d’intérêts

Nous n’avons aucun conflit d’intérêts.

## Contribution des auteurs

Tous les auteurs ont participé à la prise en charge du patient et à la rédaction du manuscrit

## Figures and Tables

**Tableau 1:** Paramàtres biologiques des Hémodialysés Chroniques (n=68)

	**Moyenne ± écart type**	**Valeur cible**
**Hémoglobinémie (g/dl)**	9,1 ± 2	3 11-12
**Férritinémie (>g /ml)**	432 ± 417	200-500
**PTH1-84 (pg/ml)**	643,4 ± 593,2	150- 300
**Calcémie (mg/l)**	89,2 ± 9	84-95
**Phosphorémie (mg/l)**	52 ± 12,4	35-55
**PAL (UI /l)**	300 ± 423	20-80
**RA (mEq/l)**	19,2 ± 2	25-28
**Kt/V**	1,58 ± 0,31	≥ 1,2
**PRU (%)**	77,7± 6,5	≥ 65
**CRP (mg/l)**	9,2 ± 5,5	< 6
**Albuminémie (g/l)**	37,8 ± 4,2	≥ 4

**Tableau 2:** Facteurs de risque liés à l’hypothyroêdie biologique chez l’hémodialysé chronique

	**Groupe 1 (n= 19) Hypothoïdie Biologique**	**Groupe 2 (n= 49) Euthyroïdie Biologique**	**P**
Age moyen (années)	51.2 ± 20,6	40.4 ± 15	3 0.003
Durée moyenne totale en dialyse (mois)	132 ± 77	105 ± 80	0.04
Diabète (%)	10, 5	4	NS
CRP (mg/l)	12.3 ± 5,2	7 ± 4,8	0.001

CRP : C-Reactive protein, NS : Non-significatif

**Tableau 3:** Profil thyroïdien évolutif des hémodialysés chroniques au cours des trois années de suivi : le nombre des hémodialysés chroniques a été ajusté en prenant en compte les décàs, les malades ayant changé le centre de dialyse (déménagement, immigration…) et les nouveaux malades recrutés (ayant bénéficié d’un examen clinique et bilan thyroïdien initial, ils sont revenus en faveur d’une hypothyroïdie clinique et biologique)

	2008 n= 65*	2009 n=60*	2010 n=57*
**FT3 basse**	**19**	**19**	**18**
**FT4 basse**	**10**	**10**	**10**
**TSH_us_ élevée**	**2**	**1**	**1**
**Incidence de l’hypothyroïdie biologique**	**0%**	**0%**	**0%**

TSHus: thyréostimuline ultra-sensible, FT3 : Triiodothyronine libre, FT4 : Thyroxine libre

**Tableau 4:** Comparaison de nos résultats par rapport à la littérature

	**n**	**Prévalence de l’hypothyroïdie (%)**
**Quion-Verde (1984)**	NP	5
**Kaplein (1988)**	287	66
**Lim (2001)**	NP	25
**Zoccali (2005)**	200	20
**Notre série (2007)**	68	28

NP: non précisé
